# Coarse Particulate Matter (PM_2.5–10_) Affects Heart Rate Variability, Blood Lipids, and Circulating Eosinophils in Adults with Asthma

**DOI:** 10.1289/ehp.9499

**Published:** 2007-01-18

**Authors:** Karin Yeatts, Erik Svendsen, John Creason, Neil Alexis, Margaret Herbst, James Scott, Lawrence Kupper, Ronald Williams, Lucas Neas, Wayne Cascio, Robert B. Devlin, David B. Peden

**Affiliations:** 1 Center for Environmental Medicine, Asthma, and Lung Biology, School of Medicine, University of North Carolina at Chapel Hill, North Carolina, USA; 2 National Health and Environmental Effects Research Laboratory, U.S. Environmental Protection Agency, Research Triangle Park, North Carolina, USA; 3 Department of Biostatistics, School of Public Health, University of North Carolina at Chapel Hill, North Carolina, USA; 4 National Exposure Research Laboratory, U.S. Environmental Protection Agency, Research Triangle Park, North Carolina, USA; 5 Division of Cardiology, Brody School of Medicine, East Carolina University, Greenville, North Carolina, USA

**Keywords:** asthma, coarse PM, heart rate variability, inflammatory markers, lipids, systemic inflammation

## Abstract

**Introduction:**

We investigated whether markers of airway and systemic inflammation, as well as heart rate variability (HRV) in asthmatics, change in response to fluctuations in ambient particulate matter (PM) in the coarse [PM with aerodynamic diameter 2.5–10 μm (PM_2.5–10_)] and fine (PM_2.5_) size range.

**Methods:**

Twelve adult asthmatics, living within a 30-mile radius of an atmospheric monitoring site in Chapel Hill, North Carolina, were followed over a 12-week period. Daily PM_2.5–10_ and PM_2.5_ concentrations were measured separately for each 24-hr period. Each subject had nine clinic visits, at which spirometric measures and peripheral blood samples for analysis of lipids, inflammatory cells, and coagulation-associated proteins were obtained. We also assessed HRV [SDNN24HR (standard deviation of all normal-to-normal intervals in a 24-hr recording), ASDNN5 (mean of the standard deviation in all 5-min segments of a 24-hr recording)] with four consecutive 24-hr ambulatory electrocardiogram measurements. Linear mixed models with a spatial covariance matrix structure and a 1-day lag were used to assess potential associations between PM levels and cardiopulmonary end points.

**Results:**

For a 1-μg/m^3^ increase in coarse PM, SDNN24HR, and ASDNN5 decreased 3.36% (*p* = 0.02), and 0.77%, (*p* = 0.05) respectively. With a 1-μg/m^3^ increase in coarse PM, circulating eosinophils increased 0.16% (*p* = 0.01), triglycerides increased 4.8% (*p* = 0.02), and very low-density lipoprotein increased 1.15% (*p* = 0.01). No significant associations were found with fine PM, and none with lung function.

**Conclusion:**

These data suggest that small temporal increases in ambient coarse PM are sufficient to affect important cardiopulmonary and lipid parameters in adults with asthma. Coarse PM may have underappreciated health effects in susceptible populations.

In a recent review article of the health effects of coarse airborne particles on health, Brunekreef and Forsberg (2005) call for special consideration in studying and regulating coarse particulate matter [PM with aerodynamic diameter 2.5–10 μm (PM_2.5–10_)] separately from fine particulate matter (PM_2.5_). Epidemiologic evidence indicates that coarse PM had as strong a short-term effect (or stronger) as fine PM on asthma, chronic obstructive pulmonary disease (COPD), cardiac, and respiratory hospital admissions (Brunekreef and Forsberg 2005; Burnett et al. 1997, 1999; Chen et al. 2004; Sheppard et al. 1999). There is a growing body of work examining the mechanisms of effect of fine PM on heart rate variability (HRV) and systemic inflammation in susceptible populations such as the elderly, individuals with COPD, and individuals with recent myocardial infarction, hypertension, diabetes, or ischemic heart disease (Chuang et al. 2005; Liao et al. 1999; O’Neill et al. 2005; Park et al. 2005; Sullivan et al. 2005; Wheeler et al. 2006). However, few if any studies have examined potential mechanisms of effect of coarse PM to explain the epidemiologic associations between increased mortality/morbidity and exposure to ambient coarse PM.

Coarse PM can be distinguished from other particulate sizes by the content of bio-active microbial products. Becker et al. have reported that coarse PM activates macrophages and monocytes *in vitro* in a toll-like receptor (TLR)2- and TLR4-dependent fashion, with a significant fraction of this biologic activity being ascribed to endotoxin (Becker et al. 2002; Soukup and Becker 2001). Alexis et al. (2006) recently showed that in healthy individuals, endotoxin on inhaled coarse PM elicits innate immune responses *in vivo* on airway macrophages. Likewise, endotoxin found in ambient PM samples (Mueller-Anneling et al. 2004), indoor dust samples (Michel et al. 1996; Pacheco et al. 2003; Thorne et al. 2005), and via personal ambient air monitoring (Rabinovitch et al. 2005) was linked to increased respiratory morbidity in children, demonstrating the likely importance of endotoxin containing coarse PM. Adachi et al. (2006) found that intraperitoneally administered endotoxin decreases heart rate variability measures such as rMSSD (root mean square of successive differences in normal-to-normal R-R intervals) and spectral density at low and high frequencies in a mouse model. Moreover, we and others have found that bronchial challenge with endotoxin also induces systemic inflammatory effects in asthmatics, even at inhaled doses that do not cause overt airway or systemic symptoms (Alexis et al. 2004; Michel et al. 1992, 1997).

Collectively, these observations led to the hypothesis that coarse PM has the ability to induce respiratory, cardiovascular, and systemic effects in humans and perhaps more so in those with preexisting airway disease such as asthma. As in our previous panel study on the effects of ambient PM on highway patrol officers (Riediker et al. 2004), we employed a repeated-measures design and examined a panel of 12 adults with asthma to determine the effects of two PM size fractions on *a*) circulating immune cells and prothrombotic factors, *b*) the induction of airway inflammation, and *c*) changes in cardiac autonomic function. Asthmatics were studied because they were considered more likely to demonstrate cardiopulmonary and systemic effects from ambient PM than healthy populations.

## Materials and Methods

### Population and study design

Twelve adults with persistent asthma (ranging from mild to severe disease), living within a 30-mile radius of the PM ambient exposure monitor located at the U.S. Environmental Protection Agency (EPA) research facility in Chapel Hill, North Carolina, were each monitored over a 12-week period. The study was designed to evaluate a range of ambient PM concentrations. Each subject made nine clinic visits, five the first week and four spaced randomly over the subsequent 6–11 weeks. Enrollment occurred from 9 September 2003 to 19 July 2004. Subjects were excluded from the study if they had a medical history of cystic fibrosis, COPD, chronic bronchitis, recurrent pneumonia, pulmonary embolism, congestive heart failure, vocal cord dysfunction, chest wall deformity, autoimmune disease, diabetes, existing heart disease, or any health problem that precluded following study protocol. Potential study subjects were excluded if they smoked more than two packs of cigarettes in the year before study enrollment. The University of North Carolina (UNC) Biomedical Institutional Review Board reviewed and approved the research protocol as did the U.S. EPA. All study subjects gave informed consent before participation.

### Study logistics

At enrollment, we obtained demographic information and medical histories using a standardized questionnaire. During the first clinic visit, participants underwent a physical exam, phlebotomy, electrocardiogram (ECG), and a pulmonary function test. The subsequent eight clinic visits included a phlebotomy, physical examination, and pulmonary function testing. Eight of 12 volunteers consented to epicutaneous skin testing during the study period. Four volunteers were unwilling to discontinue antihistamine therapy and were not skin tested. Skin prick tests (SPT) for house dust mites, German cockroach, an Eastern tree mix, grass mix, two mold mixes, guinea pig, rat, cat, and mouse were performed, with four of the eight tested subjects having a positive response to at least one allergen, three persons having completely negative skin tests, and one having invalid results due to a nonreactive histamine control test. Use of asthma medications was recorded, including anti-inflammatory controller medication (inhaled corticosteroids, oral corticosteroids, oral leukotriene inhibitors), and rescue medication (short-acting beta_2_ agonist, albuterol). Asthma severity was classified using the National Heart, Lung, and Blood Institute (NHLBI) Asthma Guidelines (NHLBI 2003). Daily asthma symptoms and medication use were recorded electronically in a personal digital assistant (PDA).

### PM air pollution measurements

Daily ambient coarse (PM_2.5–10_) and fine (PM_2.5_) size PM were measured separately for each 24-hr period using a Dichotomous Partisol-Plus Sequential Air Sampler (Model 2025-D; Rupprecht & Patashnick Co., Inc., Albany, NY) located on the roof of the U.S. EPA Human Studies Facility on the UNC campus in Chapel Hill. Coarse PM is usually not directly measured, only calculated as the difference between PM_10_ and PM _2.5_. However, we directly measured coarse PM and fine PM separately within the same air stream using a method that first separates the particles according to their size fraction before collecting them. PM mass retained on the Teflon filters (47 mm 2.0 μm; Teflo Pallflex Gelman Scientific; Pall Corporation, Ann Arbor, MI) was weighed on a microbalance in an EPA weight chamber.

### Lung function, airway inflammation, and airway cell surface marker expression

Lung function was measured at each clinic visit using a spirometer (Sensormedics Corporation, Yorba Linda, CA). Pre-bronchodilator values were used. Induced sputum was performed according to previously published procedures (Alexis et al. 2001, 2004, 2005), and airway inflammation measures included enumeration of inflammatory cells (neutrophils, eosinophils, monocytes, macrophages). Flow cytometry was performed using a FACSORT (Becton Dickinson, Franklin Lakes, NJ) as previously described (Alexis et al. 2005, 2006). Macrophages, monocytes, neutrophils, and lymphocytes in sputum were labeled with fluorescent monoclonal antibodies, identified, and gated based on light scatter properties (FSC: cell size; SSC: cell granularity) and positive expression for CD45 (panleukocyte marker), HLA-DR/CD14 (macrophages/ monocytes), CD16/FcgRIII (neutrophils), CD16-/FcgRIII (eosinophils), and CD3 (lymphocytes). The appropriate isotypic controls were used. Analysis was performed using the Cell Quest software (Becton Dickinson) and receptor expression was expressed as mean fluorescence intensity (MFI).

### Circulating cells, lipids, and proteins

During each clinic visit, blood was drawn using standardized procedures for phlebotomy, collection, and storage. Blood samples were taken from antecubital veins and collected into vacuum tubes. Blood samples were sent to LabCorp (Burlington, NC) for a complete blood count analysis that included differential leukocyte analysis.

Plasma from these samples was distributed into aliquots and stored at −80°C until assayed in our laboratory using commercially available ELISA kits. Analytes of interest included interleukin-6, (R&D Systems, Minneapolis, MN), protein C, prothrombin, plasminogen, Factor VII, Factor IX (Enzyme Research Labs, South Bend, IN), plasminogen activator inhibitor type 1 (Oncogene Science, Cambridge, MA), von Willebrand factor (Diagnostica Stago, Asnières-sur-Seine, France), and fibrinogen (DiaSorin, Stillwater, MN).

### HRV assessment using ambulatory ECG

During the first week of the study, changes in HRV parameters were measured using an ambulatory ECG (Zymed Inc., Camarillo, CA). After a 20-min rest, ECG R-waves were recorded from participants in a supine position. Subjects wore the ambulatory ECG recorders for four 24-hr periods. Each morning, at the beginning of the clinic visit, volunteers rested quietly in a supine position for 20 min followed by a specific 10-min period of recording (again in the supine position). The final 10-min period was used specifically for frequency domain analysis of HRV. Each 24-hr period of ECG data was analyzed by a cardiac electrophysiology nurse specialist and reviewed by a cardiologist. We measured ASDNN5 (the mean of the standard deviation in all 5-min segments of a 24-hr recording), SDANN5 (the standard deviation of the average of normal-to-normal intervals in all 5-min segments of a 24-hr recording), SDNN24HR [the standard deviation of all normal-to-normal R-R intervals in a 24-hr recording (milliseconds)], SDNN7min taken from the 7-min rest period each morning, and rMSSD. Additional measurements in the time domain included pNN50 [the percentage of differences between adjacent normal to normal intervals that are > 50 msec for both a 24-hr period (pNN50_24), and a 7-min period (pNN50_7), measured after 20 min supine] and the mean cycle length of normal R-R intervals (MCL). Mean heart rate (beats per minute), a general marker of autonomic function, was derived from the RR-interval record.

SDNN24HR was used to estimate the overall modulation of autonomic nervous system function and reflects total variability, whereas rMSSD estimates high-frequency variations in heart rate and primarily reflects parasympathetic activity. ASDNN5 includes respiratory-mediated parasympathetic input as well as baroreceptor-mediated sympathetic and parasympathetic input. To address the issue of HRV circadian cycles, we examined several HRV parameters from the same time each day, pNN50 measured for 7 min, and SDNN measured for a 7-min period during the 20-min rest period at the same time each morning.

We used a fast Fourier transformation to calculate the power spectral density curve. We used the area under the curve in the high-frequency range (0.15–0.40 Hz) to estimate parasympathetic modulation of variability. And we used low-frequency (LF) power (0.04–0.15 Hz) to estimate the joint contribution of parasympathetic and sympathetic influences on HRV, although it reflects primarily sympathetic modulation.

### Statistical analyses

We used linear mixed models with restricted maximum-likelihood estimation to assess potential time-varying associations of coarse and fine PM with sputum measurements, lung function, measures of HRV, proteins associated with plasma coagulation, hemostatic and inflammatory markers, and serum lipids. A 24-hr or 1-day lag of effect was assumed; the daily 24-hr PM concentrations were matched with the outcome measurements of the subsequent day. We also evaluated 2- and 3-day lags (Peel et al. 2005; Pekkanen et al. 2002; Peters et al 2004; Ruckerl et al. 2006; Timonen et al. 2006; Zeka et al. 2005) for all outcomes.

Models included the time-varying factors of atmospheric average daily temperature, humidity, and pressure. Time-invariant subject specific characteristics (such as age and sex) were not included in the final models because no differences in main effect estimates were seen with the time-invariant variables. Each subject served as his or her own control by study design. Daily PM concentrations and atmospheric variables were modeled as fixed effects.

The models included a random intercept for each subject to help account for between-subject variability. The correlation matrix structure with the repeated measures statement was specified as a spatial power function with an exponential time term of the form *f* (*d_ijj_*′) = ρ*d_ijj_*′. This parameterization accounts for the variable time between clinic visits for each individual; in particular, for the *i*th person corr(*Y_ij_*, *Y_ij_*′) = ρ*^d^^ijj^*′, where *d_ijj_*′ is the time (in days) between responses *Y_ij_* and *Y_ij_*′ for the *i*th person. All statistical computations were performed with SAS software version 8.2 (SAS Institute Inc., Cary, NC) using the “proc mixed” (mixed models) procedure. To evaluate potential outliers we examined the regression residuals. Plots of the residuals versus predicted values were constructed and examined for outliers. In addition, we also used the interactive data analysis feature in SAS to construct crude bivariate plots of coarse PM and main outcomes variables for all study subjects and for each individual. There was no evidence of significant influence by outliers or the models being driven by one or two subjects.

## Results

### Environmental measures

The mean (± SD) coarse PM concentration for the 284 days sampled was 5.3 ± 2.8 μg/m^3^ with a range of 0–14.6 μg/m^3^; for fine PM, the mean concentration was 12.5 ± 6.0 μg/m^3^ with a range of 0.6–37 μg/m^3^. The average temperature was 17.6°C, and the relative humidity 49.1%. Coarse and fine PM were not strongly correlated with relative humidity or barometric pressure, although temperature was statistically significantly correlated (0.48 and 0.61, respectively, *p* < .01). The fine PM and PM_10_ levels never exceeded the 1990 U.S. National Ambient Air Quality 24-hr standard of 65 μg/m^3^ and 150 μg/m^3^, respectively (U.S. EPA 1990). Summary statistics for air pollution and weather characteristics are presented in Table 1. Subsequent results are presented for a 1-day lag. We evaluated 2- and 3-day lags and did not find any patterns of statistically significant associations with the 2- or 3-day lags with measures of HRV, blood lipids, coagulation, or lung function and markers of inflammation.

### Subjects

The 12 subjects (3 male, 9 female) ranged in age from 21 to 50 years, with a mean of 33 years. Three subjects were African American (Table 2). Most asthmatics had mild disease severity (7 persistent, 2 intermittent), two had moderate disease and one severe. Percent predicted FEV_1_ (forced expiratory volume at 1 sec) values for the subjects ranged from 65 to 118%, with a mean of 96%. Four of 12 subjects were atopic (positive skin prick test), four were unable/unwilling to withdraw from antihistamine use but had previously tested positive for allergies before study enrollment, and four had invalid skin prick test results (negative histamine result or positive saline result) but reported allergic symptoms. All subjects took short acting beta-agonist rescue medication, and 10 of the 12 were taking controller medication (9 inhaled corticosteroids, 1 leukotriene inhibitor). Summary statistics (means, standard deviations, and range) of the HRV, circulating proteins, and lipids for the twelve study subjects are presented in the Supplemental Material Table 1 (available online at http://www.ehponline.org/docs/2007/9499/suppl.pdf). They are within the normal range of healthy individuals, and comparable to other recently published studies (Liao et al. 2004).

### Changes in spirometry, symptoms, induced sputum parameters, and total particle size fractions

No consistent associations between either coarse PM or fine PM were found with spirometric measurements, rescue beta-agonist use, or reported symptoms; nor were associations found with measures of airway inflammation [see Supplemental Material Tables 2 and 3, (including sputum macrophages, monocytes, and neutrophils) available online at http://www.ehponline.org/docs/2007/9499/suppl.pdf].

### HRV and total particle size fractions

We observed statistically significant changes in HRV associated with PM_2.5–10_ (Table 3). For a 1-μg/m^3^ increase in PM_2.5–10_, heart rate variability as measured by SDANN5, SDNN24HR, and ASDNN5 decreased 3.76% (*p* = 0.02), 3.360% (*p* = 0.02), and 0.77% (*p* = 0.05), respectively. We found a borderline association with pNN50_7min (*p* = 0.07), and no association with the 7-min SDNN (SDNN7min). High-frequency power, a measure of parasympathetic modulation, decreased 0.46% (*p* = 0.01) per 1-μg/m^3^ increase in coarse PM, indicating a decrease in vagal autonomic input to the heart associated with coarse PM. Similar patterns were not seen for fine PM (see Supplemental Material Tables 4 and 5, available online at http://www.ehponline.org/docs/2007/9499/suppl.pdf).

### Circulating proteins, cells, and lipids and total particle size fractions

Estimated regression coefficients (percent) and *p*-values for circulating proteins, cells, and lipids associated with coarse and fine PM are presented in Table 4. For a 1-μg/m^3^ increase in coarse PM, a 0.16% increase in circulating eosinophils (*p* = 0.01) was found. Ambient coarse PM was also associated with changes in blood lipids. For a 1-μg/m^3^ increase in coarse PM, an increase of 4.8% in triglycerides (milligrams per deciliter) (*p* = 0.02) and a 1.15% increase in very low-density lipoprotein (VLDL) (milligrams per deciliter) (*p* = 0.01) were found. After adjusting for ambient temperature, relative barometric pressure, and relative humidity, the association of the levels of blood coagulation–related proteins (fibrinogen and plasminogen) with coarse PM were of borderline significance (*p* = 0.07, *p* = 0.08). For a 1-μg/m^3^ increase in coarse PM, a decrease of 0.01% in plasminogen (international units per milliliter) and 0.04% in fibrinogen concentration (milligrams per milliliter) were found.

Other blood proteins and lipids revealed no associations with either size fraction of PM. Other circulating inflammatory cells (basophils, monocytes, lymphocytes, or neutrophils) were not significantly associated with coarse PM concentrations. C-reactive protein was not statistically significantly associated with increases in ambient coarse or fine PM concentrations. No consistent statistically significant relationships were seen for fine PM (Table 5, Supplemental Material, available online at http://www.ehponline.org/docs/2007/9499/suppl.pdf).

## Discussion

We examined airway and systemic responses to exposure to ambient coarse (PM_2.5–10_) and fine (PM_2.5_) PM in a cohort of adult asthmatics. Recent reports suggest that coarse PM initiates responses from airway cells *in vivo* in healthy volunteers (Alexis et al. 2006) and may be an underappreciated cause for respiratory and systemic inflammation (Brunekreef and Forsberg 2005). This study is the first to report that relatively low concentrations of coarse PM are associated with decreases in HRV, increases in circulating eosinophils, and serum triglycerides in adult asthmatics.

We were somewhat surprised that we did not observe any relationship between coarse or fine PM with rescue medication use, asthma symptoms, lung function, or airway inflammatory markers in sputum samples. However, 10 of the 12 adult asthmatics in the present study were treated with anti-inflammatory controller medication for their disease, and 9 of the 12 had mild disease. It is possible that anti-inflammatory treatment mitigated the effect of PM in their airways, or that adults with asthma are less susceptible to the effects of PM than children with asthma [in whom associations have been reported with coarse PM and increased asthma admissions to hospitals (Lin et al. 2002)]. Indeed, we have observed that inhaled corticosteroids minimizes the effect of inhaled endotoxin in asthmatics (Alexis et al. 2001). Mar et al. (2004) have reported that health outcomes associated with coarse PM were more notable in children with asthma than in adults with asthma. All the ambient fine and coarse PM concentrations in this panel study were well below the current 1997 National Ambient Air Quality Standards (U.S. EPA 1990), and the variability in PM measurements was not very large over the course of the study; this may not have been sufficient to induce acute lung inflammation in adults with well-controlled asthma. We did observe, however, a significant increase in circulating eosinophils in this cohort that was associated with coarse PM, suggesting a general pro-allergic effect of coarse PM in asthmatics, even in the absence of airway effects.

Despite the lack of short-term effect of PM on respiratory-tract biology in these asthmatics, we found that both coarse and fine PM had a significant effect on cardiac autonomic function as reflected by changes in heart rate variability. However, the associations between coarse PM and HRV were stronger and more consistent than with fine PM. In particular, greater effects were noted between coarse PM exposure and decreased high-frequency power (and percent high-frequency power) in the frequency domain, and decreased ASDNN5, SDANN5, and SDNN24HR parameters in the time domains. These measures are consistent with decreased parasympathetic influence and vagal tone. With respect to fine PM, there was a modest association with two heart rate variability parameters (SDANN5 and rMSSD).

Our HRV findings are consistent with those of Gong et al. (2004), who reported that mild asthmatic and normal volunteers undergoing a controlled exposure to particulate matter (80% of which was coarse PM by mass) had a small but significant increase in heart rate with decreased HRV in both normal volunteers and asthmatics, though the effects in normal volunteers were somewhat more pronounced. Similarly, the decreases in HRV measurements were comparable in magnitude to decreases associated with fine PM in other susceptible populations (Gold et al. 2000; Holguin et al. 2003; Liao et al. 1999, 2004; Park et al. 2005; Pope et al. 1999, 2004). For example, in our study the estimated regression coefficient for high frequency was a −4.6 [95% confidence interval (CI), −7.9 to −1.4] percent change with a 10-μg/m^3^ increase in coarse PM; Liao et al. (2004) reported an estimated regression coefficient for high frequency of 5.1 (95% CI, −8.0 to −2.1) with a 10-μg/m^3^ increase in fine PM.

There was also a near significant decrease of plasma plasminogen, fibrinogen, and von Willebrand factor (suggesting metabolic consumption of these agents), and a significant increase in triglycerides and VLDL related to increased exposure to coarse PM. Recently published clinical and epidemiologic studies support the plausibility of the increased triglycerides and VLDL association with elevated PM as a potential mechanism for atherosclerotic plaque progression (Chen and Nadziejko 2005; Kunzli et al. 2005; Sun et al. 2005; Suwa et al. 2002; Tomao et al. 2002).

Animal models and epidemiologic studies have shown an association between PM exposure and modified lipid levels. Suwa et al. (2002) demonstrated in Watanabe heritable hyperlipidemic rabbits that exposure to PM_10_ increased the total amount of lipids in aortic lesions. Sun et al. (2005) showed in a murine model that the lipid content in the aortic arch increased 1.5-fold in mice fed a high-fat chow diet and exposed to PM versus filtered air. In an epidemiologic case–control study of traffic-exposed police officers, the average values of HDL cholesterol and triglycerides were elevated in the exposed group versus an unexposed control group (Tomao et al. 2002). With respect to HRV, lipids, and other circulating markers of inflammation and PM exposure, several recent studies have begun to report an association that involves systemic inflammation as a possible mechanism underlying the association. Yue et al. (2006) found an association between abnormal HRV and blood markers of inflammation in coronary artery disease patients, and Sajadieh et al. (2004) reported earlier that reduced HRV is associated with subclinical inflammation in middle-aged and elderly subjects with no apparent heart disease. Consistent with these reports are our findings in asthmatics that ambient coarse PM is associated with increased serum triglycerides, decreased HRV, and increased circulating granulocytes (eosinophils), suggesting a complex network of interrelated pathways at work with respect to the health effects of PM exposure.

This study is one of few with daily gravimetric measurements of both the coarse and fine PM size fractions. Daily ambient fine PM and coarse concentrations were measured with a dichotomous Partisol-Plus Sequential Air Sampler at the central site for 11 months. To address potential spatial variation in coarse PM, we conducted a validation study with samplers at the site of the subject’s residence (Chen et al. 2007). The correlation between residential outdoor coarse PM mass concentrations and those obtained from the central ambient monitoring site were typically greater than *r* = 0.75. These results show that although coarse PM mass concentrations were temporally variable, they were relatively consistent spatially for distances up to 50 km (Chen et al. 2007). Thus, we are reasonably confident that for this panel study, the central-site exposure is an acceptable proxy for residential exposure of coarse PM. Fine PM measured at a central site is recognized as a good proxy for personal exposure (Koutrakis et al. 2005; Sarnat et al. 2000; Williams 2003a, 2003b). The recent Williams et al. studies (2003a, 2003b), conducted in Research Triangle Park, North Carolina, over the course of a 1-year period, indicate that fine PM concentrations are highly homogeneous with respect to mass at distances approaching 70 km.

Given that coarse PM is rich in biologic material, particularly endotoxin, it may not be surprising that we found effects of ambient coarse PM exposure that mimic those seen with systemic endotoxin challenge. Intravenous challenge with endotoxin is associated with decreased HRV (associated with increased risk for cardiac events), systemic inflammation, and increases in serum triglycerides and VLDL in human volunteers and in animal models (Godin et al. 1996; Goldstein et al. 1995; Hardardottir et al. 1995; Hudgins et al. 2003; Levels et al. 2003; Voss et al. 2004). We and others have found that bronchial challenge with endotoxin induces systemic inflammatory effects as well, even at inhaled doses that do not cause overt airway or systemic symptoms (Alexis et al. 2004; Michel et al. 1992, 1997). We have also observed reduced airway cytokine and macrophage responses in healthy volunteers when they were exposed to coarse PM that had been heated to deactivate biologic agents (including denature endotoxin) versus PM that had not been heated (Alexis et al. 2006). These observations are consistent with our hypothesis that persons with chronic inflammatory diseases of the airway have increased responsiveness to biologic materials contained in coarse particulates.

Our panel study design allowed us to examine both low-level ambient PM exposures and their associated daily variability while at the same having repeated intensive clinical monitoring that would be impossible in field epidemiologic studies. In summary, we found that in repeated measures on a panel of 12 well-controlled asthmatics, 1-day lagged 24-hr concentrations of ambient coarse PM were significantly associated with decreased HRV, increased circulating eosinophils, and increases in serum triglycerides, indicating that coarse PM may play an underappreciated role in pollutant-induced cardiovascular events, even in asthmatics using anti-inflammatory therapy. We also report that neither low levels of ambient coarse nor fine PM had an effect on respiratory symptoms, airway inflammation, or lung function. Further study is needed to identify the mechanisms of effect of coarse and fine PM on systemic endpoints in healthy and susceptible populations as well as the role of endotoxin and other biologic components of coarse PM on these outcomes.

## Figures and Tables

**Table 1:** Ambient air characteristics and PM concentrations (*n* = 284 days).

			Spearman ρ correlation coefficients	
Variable	Concentration (mean ± SD)	Range	PM_2.5–10_	PM_2.5_
PM_2.5–10_ (coarse)	5.3 ± 2.8	(0 to 14.6)	1	0.46*
PM_2.5_ (fine)	12.5 ± 6.0	(0.6 to 37.1)	0.46*	1
PM_10_ (total)	17.5 ± 7.8	(1.4 to 45.6)	0.73*	0.90*
Temperature (°C)	17.6 ± 8.5	(−5.0 to 32.9)	0.60*	0.48*
Relative humidity (%)	49.1 ± 14.5	(10.5 to 91.6)	0.13**	0.16
Barometric pressure (mm Hg)	756.1 ± 5.9	(741 to 770)	0.11	−0.01

* *p* < 0.01.

** *p* < 0.05.

**Table 2:** Demographic characteristics of study participants (*n* = 12).

Category	No. (%)^a^
Sex	
Female	9 (75)
Race/ethnicity	
African American	3 (25)
White	7 (58)
Latino	1 (8)
Asian	1 (8)
NHLBI severity classification	
Severe	1 (8)
Moderate persistent	2 (17)
Mild persistent	7 (58)
Mild intermittent	2 (17)
Asthma medication	
Antiinflammatory controller medication	10 (83)
Inhaled corticosteroids	
Fluticasone/salmeterol	5 (42)
Budesonide	3 (25)
Fluticasone	1 (8)
Leukotriene inhibitor (montelukast)	1 (8)
Beta-agonists (albuterol)	12 (100)
Allergies	
Antihistamine therapy	4 (33)
Subjects SPT	8 (67)
SPT positive	4 (33)
Symptoms, SPT negative	3 (25)
Uninterpretable SPT	1 (8)
IgE concentration (IU/mL)	166.7 (3–755)
Percent predicted FEV1 [%, mean (range)]	0.96 (65–118)
Age [years, mean (range)]	33.17 (21–50)

a Percentages are rounded to the near whole number.

**Table 3:** Change in HRV indices^a^ per 1-μg/m^3^ increase in PM_2.5–10_ and PM_2.5_.

	PM_2.5–10_				PM_2.5_			
	Estimated regression coefficient	SE	*p*-Value	95% CI	Estimated regression coefficient	SE	*p*-Value	95% CI
HRV								
Max heart rate	−1.95	0.88	0.03	−3.67 to −0.23	0.40	0.43	0.36	−0.45 to 1.24
ASDNN5	−0.77	0.37	0.05	−1.50 to −0.04	−0.07	0.15	0.63	−0.37 to 0.22
SDANN5	−3.76	1.53	0.02	−6.76 to −0.76	1.66	0.65	0.02	0.39 to 2.93
SDNN24HR (msec)	−3.36	1.38	0.02	−6.06 to −0.65	1.16	0.58	0.06	0.02 to 2.29
rMSSD	−0.75	0.53	0.16	−1.79 to 0.28	0.53	0.20	0.01	0.14 to 0.91
pNN50_24hour	−0.50	0.27	0.07	−1.03 to 0.03	−0.06	0.11	0.58	−0.27 to 0.15
pNN50_7min	−1.88	0.55	0.07	−2.95 to −0.81	0.47	0.42	0.27	−0.35 to 1.29
Low-frequency power	−0.19	0.42	0.65	−1.01 to 0.63	−0.23	0.14	0.11	−0.51 to 0.05
Percent low frequency	0.57	1.08	0.60	−1.55 to 2.69	−0.78	0.41	0.07	−1.59 to 0.03
High-frequency power	−0.46	0.17	0.01	−0.79 to −0.14	0.14	0.07	0.07	−0.01 to 0.28
Percent high frequency	−2.14	0.94	0.03	−3.98 to −0.30	0.64	0.36	0.09	−0.07 to 1.34

Abbreviations: Max, maximum; pNN50_24hour and pNN50_7min, percentage of differences between adjacent normal-to-normal intervals that are > 50 msec for either 24 hr or 7 min during resting period each morning.

a Adjusted for relative temperature, pressure, and humidity.

**Table 4:** Change in circulating proteins and hematologic and lipid indices^a^ per 1-μg/m^3^ increase in PM_2.5–10_ and PM_2.5_.

	PM_2.5–10_				PM_2.5_			
Outcome	Estimated regression coefficient	SE	95% CI	*p*-Value	Estimated regression coefficient	SE	95% CI	*p*-Value
Blood lipids								
Triglycerides	4.78	2.02	0.81 to 8.74	0.02	−0.63	0.84	−2.29 to 1.02	0.46
VLDL	1.15	0.44	0.29 to 2.02	0.01	−0.17	0.22	−0.61 to 0.26	0.44
Total cholesterol	0.78	0.54	−0.28 to 1.84	0.15	−0.06	0.22	−0.49 to 0.36	0.77
Hematologic factors and circulating immune cells								
Circulating eosinophils	0.16	0.06	0.04 to 0.28	0.01	−0.02	0.00	−0.02 to −0.02	0.27
Platelets	−1.71	1.11	−3.89 to 0.47	0.13	−0.01	0.45	−0.88 to 0.86	0.98
Circulating proteins								
Plasminogen	−0.01	0.01	−0.02 to 0.00	0.08	0.00	0.00	−0.01 to 0.00	0.82
Fibrinogen	−0.04	0.02	−0.08 to 0.00	0.07	0.00	0.01	−0.01 to 0.02	0.59
Von Willibrand factor	−1.23	0.66	−2.53 to 0.06	0.07	−0.31	0.29	−0.87 to 0.25	0.28
Factor VII	−0.90	0.85	−2.58 to 0.77	0.29	−0.65	0.33	−1.29 to −0.01	0.05

a Adjusted for relative temperature, pressure, and humidity.
